# A Comprehensive View of the Epigenetic Landscape. Part II: Histone Post-translational Modification, Nucleosome Level, and Chromatin Regulation by ncRNAs

**DOI:** 10.1007/s12640-014-9508-6

**Published:** 2014-12-17

**Authors:** Anna Sadakierska-Chudy, Małgorzata Filip

**Affiliations:** 1 Laboratory of Drug Addiction Pharmacology, Institute of Pharmacology Polish Academy of Sciences, Smetna 12, 31-343 Kraków, Poland; 2 Department of Toxicology, Faculty of Pharmacy, Jagiellonian University Medical College, Medyczna 9, 30-688 Kraków, Poland

**Keywords:** Chromatin cross-talk, Histone code, Histone post-translational modifications, Nucleosome positioning, Histone tail clipping, ncRNAs

## Abstract

The complexity of the genome is regulated by epigenetic mechanisms, which act on the level of DNA, histones, and nucleosomes. Epigenetic machinery is involved in various biological processes, including embryonic development, cell differentiation, neurogenesis, and adult cell renewal. In the last few years, it has become clear that the number of players identified in the regulation of chromatin structure and function is still increasing. In addition to well-known phenomena, including DNA methylation and histone modification, new, important elements, including nucleosome mobility, histone tail clipping, and regulatory ncRNA molecules, are being discovered. The present paper provides the current state of knowledge about the role of 16 different histone post-translational modifications, nucleosome positioning, and histone tail clipping in the structure and function of chromatin. We also emphasize the significance of cross-talk among chromatin marks and ncRNAs in epigenetic control.

## Introduction

Over the last decade, researchers worldwide have revealed a huge amount of information about the epigenome, but many questions still remain unanswered. Epigenetic studies focus mainly on the investigation of DNA methylation, histone variants, and histone modifications as well as nucleosome positioning. In addition, DNA- and histone-binding proteins that influence chromatin structure and non-coding RNA (ncRNA) molecules have emerged as key players in chromatin remodeling. Post-translational modifications (PTMs) of the histone tails regulate two opposite processes, namely transcriptional activation and repression (Cohen et al. 2011). In addition, histone-modified tails serve as binding and signaling platforms for regulatory and remodeling proteins, thus influencing chromatin organization (Cohen et al. 2011).

Chromatin is not static but changes according to the regulatory cue including histone-modifying, histone modification-recognizing, and histone modification-erasing proteins, so-called writer, reader, and eraser proteins, respectively. Although nucleosomes themselves are stable with limited mobility, some remodeling complexes may mobilize and/or eject the nucleosome to regulate access to DNA (for a review, see Saha et al. 2006). The clipping of histone tails can affect the recruitment of various factors affecting downstream processes (Azad and Tomar 2014). It is worth mentioning that the removal of the N-terminal tail of histones also influences structure and dynamics of chromatin that could promote or inhibit transcription activity (Bannister and Kouzarides 2011). On the other hand, histone tails may serve as docking sites for regulatory proteins that promote gene transcription (Martin and Zhang 2005). An increasing body of evidence suggests that ncRNAs (including miRNA, siRNAs, asRNAs, piRNA, and lncRNAs) can play a role in the regulation of chromatin state and gene expression (Kaikkonen et al. 2011; Gomes et al. 2013).

In this review, the present knowledge of various histone PTMs, nucleosomes, chromatin-modifying enzymes, and ncRNAs is summarized to improve the understanding of complex genetics and epigenetic interactions.

## Histone Level

The structure of chromatin determines the accessibility of DNA to transcriptional machinery; thus, it is closely related to gene activity. The N-terminal and C-terminal tails of histones undergo reversible PTMs that change their interaction with DNA and serve as “docking stations” for nuclear proteins. These modifications include methylation, acetylation, phosphorylation, glycosylation, carbonylation, ubiquitylation, biotinylation, sumoylation, citrullination, ADP-ribosylation, N-formylation, crotonylation, propionylation, and butyrylation, as well as proline and aspartic acid isomerization. Histone lysine methylation appears to be relatively stable, the half-lives ranging from several hours to days (Zee et al. 2010; Huang et al. 2013a). Histone methylation can be involved in the epigenetic memory of the transcriptional status by changing chromatin organization in a mitotically heritable manner (Cazzanelli et al. 2009; D’Urso and Brickner 2014). Conversely, acetylation and phosphorylation are more dynamic, with half-lives ranging from minutes to a few hours (Jackson et al. 1975; Zheng et al. 2013). These changes are related to the open structure of chromatin. The list of reversible PTMs is shown in Table 1. The histone tails are readily accessible to various enzymes, including histone methyltransferases (HMTs), histone acetyltransferases (HATs), histone deacetylases (HDACs), and kinases. The histone-modifying enzymes can add or remove covalent modifications and are called “writers” and “erasers,” respectively. Proteins that are able to interpret the histone code are known as “readers” (e.g., the PHD finger) (Musselman and Kutateladze 2009). For more details, see the “Cross-talk of chromatin marks” paragraph.

**Table 1:** The reversible histone post-translational modifications

Amino acid residue [single-letter code]	Modification	References
Lysine [K]	Acetylation	Kouzarides (2007)
	Mono-, di-, and trimethylation	
	Ubiquitination	Wilkinson (2000)
	Biotinylation	Ballard et al. (2002)
	Sumoylation	Iñiguez-Lluhí (2006)
Arginine [R]	Symmetric mono- and di-methylation asymmetric mono- and di-methylation	Chang et al. (2007)
	Mono-and poly-ADP-ribosylation	Koch-Nolte et al. (2008)
Serine [S]	Phosphorylation	Bannister and Kouzarides (2011)
Tyrosine [Y]	Phosphorylation	
Threonine [T]	Phosphorylation	
Glutamate [E]	Mono- and poly-ADP-ribosylation	Koch-Nolte et al. (2008)
Proline [P]	Isomerization	Iñiguez-Lluhí (2006)

Epigenetic modifications constitute a set of tags, which reflect the local state of chromatin, and are defined as the “histone code.” All marks act as signaling platforms and govern interactions of DNA and histones with other proteins, leading to activation and repression of transcription depending on the nature and position of the modifications (Strahl and Allis 2000; Jenuwein and Allis 2001). To date, at least fifteen types of PTMs have been identified at 130 different sites on core (histones) and linker histones.

## The Nomenclature for Modified Histones

The Brno nomenclature was created by a consortium of European laboratories to standardize the notation for histones (Turner 2005). An example of this notation is shown below, the nomenclature starts from the histone protein (H3, H4, H2A, H2B, or H1), then the modified amino acid residue (i.e., “K4” representing lysine 4), and finally the type of modification (i.e., “me2” represents di-methylation). 

The lowercase letters for modifications help to distinguish them from amino acids or histones (e.g., H2A), so that the use of commas or dots to separate the individual modified residues is not necessary. Multiple modifications in the same tail of a histone can be listed sequentially, for example, H3K4me3K9acS10ph. When the modified residue is unknown, the modification should follow the histone (e.g., H4ac or H2Bar1). When an unmodified residue is essential for epigenetic interactions, it should be inserted without additions (e.g., H3K9S10ph, lysine 9 is unmodified and serine 10 is phosphorylated). The proposed nomenclature for known histone PTMs presented in this review can be found in Table 2.

**Table 2:** The proposed nomenclature for histone modifications

Type of modification	Amino acid (symbol)	Level of modification	Abbreviation of modifications	Examples of notion
Methylation	Arginine (R) Arginine (R) Arginine (R) Lysine (K) Lysine (K) Lysine (K)	Mono- Di-, symmetrical Di-, asymmetrical Mono- Di- Tri-	me1 me2s me2a me1 me2 me3	H3R2me1 H2ARme2s H4R3me2a H3K9me1 H3K9me2 H3K9me3
Acetylation	Lysine (K)	Mono-	ac	H4K5ac
Phosphorylation	Serine (S) Threonine (T) Tyrosine (Y)	Mono- Mono- Mono-	ph ph ph	H3S10ph H3T11ph H3Y41ph
Ubiquitination	Lysine (K) Lysine (K) Lysine (K)	Mono- Di- Poly-	ub1 ub2 ubn	H2AK119ub1 H2AK119ub2 H2AK119ubn
Sumoylation	Lysine (K)	Mono-	su	H4K14su
Biotynylation	Lysine (K)	Mono-	bio	H2AK9bio
Citrullination	Arginine (R)	Mono-	cit	H3R17cit
ADP-ribosylation	Glutamate (E) Arginine (R) Glutamate (E)	Mono- Mono- Poly-	ar1 ar1 arn	H1E15ar1 H1.3R33ar1 H2BE2arn
β-N-glycosylation	Serine (S) Threonine (T)	Mono- Mono-	glc glc	H3T32glc H3S10glc
Isomerization	Proline (P) Aspartic acid (D)	*cis*/*trans*	iso iso	H3P38iso H2BD25iso?
Crotonylation	Lysine (K)	Mono-	cr	H2BK5cr
Formylation	Lysine (K)	Mono-	fo	H1K17fo
Propionylation	Lysine (K)	Mono-	prop	H3K23prop
Butyrylation	Lysine (K)	Mono-	buty	H4K5buty

## Post-translational Modifications of Histones

### Methylation

Histone methylation involves transfer of a methyl group from the high-energy enzymatic donor SAM to ε-amino groups of lysine and arginine mainly on the H3 and H4 tails. In contrast to acetylation, methylation is more complex and induces structural changes. Each methyl moiety adds 14 Daltons to the histone protein and influences chromatin folding via an electrostatic mechanism (Völkel and Angrand 2007). As mentioned previously, lysine methylation is relatively stable, whereas arginine methylation is temporary; the attachment of methyl groups is carried out by arginine methyltransferases and the removal by JMJD6 demethylase (Chang et al. 2007; Bassett and Barnett 2014). Additionally, methylarginine can be converted to citrulline by specific deiminase (for more details, see the paragraph “Citrullination”). Methylation can lead to activation or silencing of gene expression depending on its localization in the histone tail. Moreover, the effect is subject to the degree of methylation of the amino acid residue. Lysine can be mono-, di-, or trimethylated by HMTs (Kouzarides 2007).

In general, transcription activity is associated with methylation of H3 lysine 4, 36, and 79, but methylation of H3 lysine 9 and 27, as well as H4K20, is linked to the repressed state. H3K4me3 accumulates predominantly at the 5′-end of active genes and promotes transcription by interaction with RNA polymerase II (RNA pol II). In contrast, H3K36me3 tends to accumulate at the 3′-end of genes and interacts with RNA pol II, supporting elongation (Mas et al., book 2011). On the other hand, H3K9me2/me3 and H4K27me2/me3 are connected with heterochromatin formation and silencing of gene expression. Interestingly, H3K9 methylation creates a docking site for corepressive heterochromatin protein 1 (HP1) family (isoforms HP1-α and HP1-β) that may increase chromatin compaction (Font-Burgada et al. 2008). Vakoc et al. (2005) demonstrated that H3K9me3 and HP1γ were associated with transcribed regions of the genome and, together with other histone marks (H3K4, H3K36, and H3K79), were responsible for transcriptional elongation. This finding indicates a dual role of H3K9 methylation in repressed and active states of chromatin and provides insights into the complex nature of the histone code.

Until recently, lysine methylation was considered a stable, long-term epigenetic modification, but the identification of histone demethylase (HDM) enzymes completely changed this view. Lysine methylation and demethylation are controlled by HMTs and HDMs, respectively. Most histone lysine methyltransferases contain catalytic domains referred to as the SET domain, and they modify virtually all lysines except those modified by the Dot/DOT1L1 family, which methylates H3K79 (Ng et al. 2002; Van Leeuwen et al. 2002). HDMs belong to two families, the LSD1 family and the JmJC domain-containing family. These enzymes can recognize specific lysine residues and distinguish between the mono-, di-, and trimethylation states (Klose et al. 2007).

Like lysine, arginine can be mono- and di-methylated either symmetrically or asymmetrically on histone H3 at R2, R17, and R26 and on histone H4 at R3 (Lee et al. 2005a). At this point, two mammalian types of arginine methyltransferase have been identified. Type I includes protein arginine methyltransferases: PRMT1, PRMT2, PRMT3, PRMT6, PRMT8, and cofactor-associated arginine methyltransferase 1 (CARM1, referred to as PRMT4), which generate monomethyl or asymmetric dimethylarginine (McBride and Silver 2001; Yang and Bedford 2013). The type II protein arginine methyltransferases: PRMT5 (also called JBP1), PRMT7, and PRMT9, which create monomethyl and symmetric dimethylarginine (Pal et al. 2004; Lee et al. 2005b; Cook et al. 2006). PRMT1 methylates histone H4 at arginine 3 (R3) and histone H2A at R3. CARM1 methylates arginine 2, 17, and 36 at the N-terminus of histone H3 and arginine 128, 129, 131, and 134 at the C-terminus of histone H3 as well as histone H2A. PRMT5 methylates histone H3 at R8 and histone H4 at R3 (Schurter et al. 2001). Methylation at arginine residues within histones can be dynamically regulated (e.g., during oogenesis and early development) (Wysocka et al. 2006), and therefore may affect the chromatin structure and transcriptional activity (see review Litt et al. 2009). It has been established that asymmetric dimethyl modifications of histone H3R17 and H4R3 are associated with active chromatin; by contrast, symmetric dimethyl modifications of histone H3R8 and H4R3 are repressive marks of chromatin (Di Lorenzo and Bedford 2011). Interestingly, Miao et al. (2006) suggested that (the) methylation of histone H3 at arginine 17 (H3R17) regulates promoters of inflammatory genes and thus may play a role in inflammatory diseases.

### Acetylation

The N-acetylation of lysine residues in histones H3 and H4 is mediated by HATs, which require acetyl-CoA as a coenzyme to catalyze the reaction (Kouzarides 2007; Berndsen and Denu 2008). Acetyl-CoA, a member of the high-energy CoA compounds, is the substrate used by acetyltransferases to catalyze the lysine acetylation reaction (Chen et al. 2007). Generally, acetylation of histones is associated with remodeling chromatin organization for transcriptionally active regions of chromatin. Acetyl groups (COCH_3_) are transferred to lysines on N-terminal tails of histones by HATs. Acetylation removes the positive charge of lysine, causing chromatin relaxation and ultimately facilitating the access of transcription factors to gene promoters (Allfrey 1966). This process can be influenced by other PTMs, for example, H3S10 phosphorylation can stimulate acetylation of histone H4K14 supporting transcription activity (Lo et al. 2000). There is also a relationship between histone acetylation and H3 methylation (Delcuve et al. 2012). Lysine acetylation is a reversible modification of histone proteins that play a significant role in regulating gene expression. The predominant acetylation sites are lysine 5 and 9 of histone H2A; lysine 5, 12, 15, and 20 of histone H2B; 9, 14, 18, and 23 of histone H3; and lysine 5, 8, 12, 16, and 20 of histone H4 (Turner 2002; Kallin and Zhang 2004). In turn, HDACs remove the acetyl groups leading to hypoacetylation (restoration of positive charge), chromatin compression, and, consequently, inhibition of transcription activity.

### Phosphorylation

Phosphorylation is a highly dynamic modification and can occur on serine (S), threonine (T), and tyrosine (Y) residues in four core histones (H2AS1; H2A.XS139; H2BS14; H3S10, S28, T3, T11, T32, and H4S1), mainly in the N-terminal tails (Pawlak and Deckert 2007; Pérez-Cadahia et al. 2010; Caperta et al. 2008). However, non-receptor tyrosine kinase JAK2 can phosphorylate the core regions, for example, tyrosine 41 on histone H3 (Dawson et al. 2009). The phosphate (PO_4_) group is added to the histone tails by various specific kinases and removed by phosphatases. Addition of the negatively charged phosphate group can induce changes in the chromatin structure. Histone phosphorylation can control several processes, including mitosis (H3S10, S28), meiosis and the DNA damage response (H4S1, H2A.XS139), and apoptosis (H2BS10), as well as gene expression (H3S10, T11) (Cheung et al. 2000; Metzger et al. 2008; Singh and Gunjan 2011). This modification is cell cycle dependent, and during mitosis, it promotes chromosomal condensation and segregation. In eukaryotes, DNA double-strand breaks (DSBs) lead to rapid phosphorylation of serine 139 at the C-terminus of histone H2A.X by PI3K kinase resulting in gamma-H2A.X (γH2A.X) (Kuo and Yang 2008). Phosphorylation of H2A.X at S139 is the first step in recruiting and localizing DNA repair proteins, including ataxia telangiectasia mutated (ATM), ATM-Rad3-related (ATR), and DNA-dependent protein kinase (DNA-PK) (Kuo and Yang 2008). It has been shown that γ-H2A.X is a sensitive and early indicator of DSBs in vitro and in vivo (Rogakou et al. 2000), and γ-H2A.X can be useful as a sensitive and early indicator of even low levels of DNA damage (Banáth et al. 2004). Most recently, it was revealed that histone H2A.X and H3 can be phosphorylated on a tyrosine residue. These phosphorylations play an important role in the DNA damage response (H2A.XY142), histone turnover (H3Y99), and chromatin structure and oncogenesis (H3Y41) (Xiao et al. 2009; Cook et al. 2009; Krishnan et al. 2009; Singh et al. 2009; Dawson et al. 2009). It is interesting that DNA damage induces dephosphorylation of threonine 142 in histone H2A.X with concurrent phosphorylation H2A.XS139, and this dephosphorylation is required for γH2A.X accumulation (Cook et al. 2009).

The most thoroughly characterized histone phosphorylation site is serine 10 on histone H3. During interphase, phosphorylation of H3S10 is mediated by two kinases: Rak2 (ribosomal S6 kinase 2) and Msk1 (mitogen- and stress-activated kinase 1), and this modification is associated with activation of numerous genes (Hartzog and Tamkun 2007). The study carried out by Nowak and Corces (2000) suggests that H3S10 phosphorylation might play a crucial role in transcription by RNA polymerase II (Pol II). Moreover, phosphorylation of H3S10 is responsible for disrupting the HP1–H3K9me3 interaction due to the reduction in affinity binding of HP1 chromodomain (Fischle et al. 2005; Sawicka and Seiser 2014). Thus, the phospho-methyl switch mechanism could explain how histone phosphorylation at S10 might induce changes in binding of effector proteins to PTMs. Interestingly, phosphorylation of histone H3 at serine 10 seems to be a response to environmental factors. Crosio et al. (2000) demonstrated that pulses of light induced changes in the distribution of phosphorylated H3S10 in the suprachiasmatic nucleus of rats, which correlated with the activation of immediate-early response genes (*c*-*fos* and *c*-*jun*). Similarly, neuronal activation by agonists of dopamine, muscarinic acetylcholine, and ionotropic glutamatergic receptors influenced the distribution of S10-phosphorylated histone H3 and gene expression in hippocampal neurons (Crosio et al. 2003).

### β-N-glycosylation

More than 500 nucleocytoplasmic proteins undergo *O*-glycosylation, which results in the monosaccharide *β*-*N*-acetylglucosamine (*O*-*β*-GlcNAc) becoming *O*-linked to the hydroxyl group of serine or threonine residues. Two evolutionarily conserved enzymes are involved in this post-translational modification, a transferase (*O*-linked *β*-*N*-acetylglucosamine transferase, alternatively *O*-GlcNAc transferase or OGT) and a hydrolase (*O*-linked *β*-*N*-acetylglucosaminidase, alternatively *O*-GlcNAcase or OGA). Interestingly, these enzymes are more highly expressed in the pancreas and brain than in other tissues (Alfaro et al. 2012). OGT catalyzes the transfer of the sugar from the donor substrate, uridine diphosphate (UDP)-GlcNAc, to the histone tails, whereas OGA removes *O*-linked *β*-N-acetylglucosamine. The *O*-GlcNAc modification appears to be highly dynamic and responds to hormones, nutrients, and cellular stress. It therefore may contribute to many diseases, including diabetes, neurodegeneration, and cancer (Slawson et al. 2005). The donor substrate, UDP-GlcNAc, is nutrient sensitive, and its intracellular concentrations correlate with glucose levels (Slawson et al. 2010).

Kim et al. (1997) first observed the *O*-GlcNAc modification in histones H1, H2A, H2B, and H3 in mouse liver and calf thymus (Sakabe et al. 2010). A recent study has revealed that all four core histones are modified with *O*-GlcNAc at specific sites, but the precise sites on histone H3 have not yet been identified (Sakabe et al. 2010). Sakabe et al. (2010) mapped three sites on histones: H2AT101, H2BS36, and H4S47. *O*-GlcNAcylation at serine 36 of H2B and at serine 47 of H4 could regulate histone tail dynamics because these amino acids are located on the lateral surface of the histone octamer in close proximity to the DNA. Furthermore, other lines of evidence indicate that serine 10 of histone H3 (Zhang et al. 2011) and serine 112 of histone H3 are also *O*-GlcNAcylated (Fujiki et al. 2011). The most recent study has identified the novel *O*-GlcNAc site in histone H3 at threonine 32 (H3T32glc) by mass spectrometry (Fong et al. 2012). This group reported that *O*-GlcNAcylation at threonine 32 on histone 3 decreases mitosis-specific phosphorylation of T32, S28, and S10 on histone H3. It is possible that the T32glc coils the tail of histone H3 to block access to the Aurora B kinase (Fong et al. 2012). The cycling distortion of *O*-GlcNAc during mitosis leads to severe cytokinesis defects, and it seems that this modification could be a target for chemotherapeutic agents (Slawson et al. 2005). The H3T32glc may be an interesting chromatin marker contributing to metabolism and insulin signaling. Moreover, ChIP-seq studies revealed that proteins binding *O*-GlcNAcylated chromatin regulate transcription of genes associated with metabolism and aging (Love et al. 2010). More recently, it was shown that TET2 and TET3 interact with OGT and target it to chromatin (Chen et al. 2013). Interestingly, the TET2/3-OGT complex co-localizes on chromatin at active promoters, initiates GlcNAcylation and influences on H3K4me3 via the methyltransferase SET1/COMPASS complex (Deplus et al. 2013).

### Carbonylation

Non-enzymatic histone post-translational modifications may play a pivotal role in regulating chromatin structure and function as well as maintaining genome stability. Protein-bound carbonyl groups are formed by direct oxidation of amino acid residues, inter alia, by reactive oxygen species (Sharma et al. 2006). These modifications mainly occur in basic amino acid residues, including arginine and lysine. Carbonylation in histone proteins may mask the positive charges and thus affect the relaxation of chromatin and accumulation of transcription factors.

Little is known about histone carbonylation, which may be related to age and environmental factors. One of the many possible explanations is that the half-life of histones within non-proliferating cells ranges between 4 and 5 months (Commerford et al. 1982), and carbonylation is not a reversible modification and therefore can accumulate in histones.

Wondrak et al. (2000) demonstrated that linker histone H1 is preferentially carbonylated in vivo because H1 is more accessible than the core histones. Another report by Goto et al. (2007) showed that histones H1, H2A, H2B, and H3 (but not H4) are carbonylated in vivo. The latter study also showed that the level of histone carbonylation was higher in the livers of younger rats than in older rats. Moreover, restriction diets in older animals led to an increase in carbonylation that was comparable to the level in younger rats. It is unclear why the histone carbonylation increase occurs during caloric restriction, which reduces oxidative stress. It is suggested that lower carbonylation in older animals may be related to the replacement of highly carbonylated molecules by less carbonylated molecules during metabolic and/or cellular turnover (Goto et al. 2007). Garcia-Gimenez et al. (2012) have revealed high levels of carbonylation on histones H1, H1^0^, and H3.1 dimers during S phase of the cell cycle. These findings provide new insights into the role of histone carbonylation in transcription, replication, and repair activities.

### Citrullination (Deimination)

Citrullination involves the conversion of peptidyl arginine to citrulline by the enzyme peptidylarginine deiminase 4 (PADI4, also known as PAD4) (Bannister and Kouzarides 2011). This reaction results in the positive charge neutralization of arginine because citrulline is neutral. PADI4 is also able to convert monomethyl arginine to citrulline; thus, arginine methylation seems to be reversible. During this process, a methyl group is removed together with the imine group of arginine (Denis et al. 2009). According to Denis et al. (2009), PADI4 binds to HDAC1, and the presence of this complex correlates with the acquisition of citrulline, histone deacetylation, and disassociated RNA polymerase II. This finding indicates that PADI4 collaborates with HDAC1 in gene silencing. Thus, citrullination may lead to transcriptional repression, but little is known about the precise mechanism of action. In contrast, a growing body of evidence links PADI enzymes to chromatin activities. Most likely, PADI4 catalyzes the citrullination of histone H4 at arginine 3 (Wang et al. 2004), while PADI2 (localized in the nucleus) appears to target histone H3 (Cherrington et al. 2010). A recent study suggests that histone citrullination may play an important role in facilitating gene expression in early embryos by creating a “platform” for HAT assembly leading to the enhancement of histone acetylation (Kan et al. 2012). It was observed that citrullination of H3R8 in patients suffering from multiple sclerosis (MS) was enriched in cytokine genes, whereas recruitment of HP1α to the promoter was significantly reduced (Sharma et al. 2012). In fact, in MS patients, activation of T cells is associated with increased expression of inflammatory cytokines (Imitola et al. 2005). In this context, citrullination of H3R8 emerges as a histone modification that affects gene silencing via HP1α. One possible explanation is that this modification reduces the affinity of the chromodomain of the HP1 proteins to the methylated lysine 9 of histone H3 resulting in a reduction in intranucleosomal bridging.

### Ubiquitylation (Ubiquitination)

Ubiquitin (Ub) is a small protein of 76 amino acids, highly conserved in eukaryotes. Ubiquitylation is one of the PTMs that rely on covalently attaching one (mono-Ub) or more ubiquitin (poly-Ub) moieties through an isopeptide bond between its C-terminal glycine and the ε-amino group of a lysine residue (Zhang 2003). The sequential action of three enzymes, E1-activating, E2-conjugating, and E3-ligating, is required for the addition of an ubiquitin moiety. Ubiquitylation is reversible, and the removal of Ub is achieved by enzymes called isopeptidases (Wilkinson 2000). Histone proteins can also be modified by ubiquitylation of the specific lysine residues K119 and K120 in histone H2A and H2B, respectively (Nickel and Davie 1989; Robzyk et al. 2000). Approximately 5–15 % of H2A and 1–2 % of H2B are ubiquitylated in higher eukaryotic organisms. The majority of H2A is monoubiquitylated, but it also can be polyubiquitylated (Nickel and Davie 1989), while H2B is only monoubiquitylated (Zhang 2003). Ubiquitylated lysine 91 on histone H4 has been identified (Yan et al. 2009), and this modification has also been discovered on histone H3 and H1, but no specific site has been defined (Zhang 2003).

The role of ubiquitylation in transcription regulation (i.e., activation or repression) is still controversial because different studies provide contradictory findings. On the one hand, H2B ubiquitylation may participate in transcriptional activation by facilitating H3K4 methylation and transcriptional elongation (Zhang 2003). On the other hand, ubiquitylation of H2A at lysine 119 is related to transcriptional repression through subsequent binding of the polycomb repressive complex (PRC) (Hunt et al. 2013). Whether histone ubiquitylation regulates gene expression in a positive or negative fashion most likely depends on its genomic location and the context of histone modifications. For example, ubiquitylation on H2BK123 in budding yeast (corresponding to H2BK120 in humans) is necessary for methylation of H3K3 and H3K79 (Sun and Allis 2002). In addition, Seigneurin-Berny et al. (2001) have shown that the murine HDAC6 binds to ubiquitin, which may suggest a relationship between ubiquitylation and acetylation. It is plausible that histone ubiquitylation can influence the folding of chromatin due to the proximity of H2AK119 to the linker histone (Bonner and Stedman 1979; Zhang 2003).

Numerous studies have confirmed that histone ubiquitylation is involved in DSB repair and the DNA damage response (Deem et al. 2012). The polyubiquitin chains on H2A are responsible for recruiting repair proteins, including BRCA1, to sites of DNA repair (Deem et al. 2012), and H2A.X and H2B ubiquitylation promotes DSB repair (Moyal et al. 2011). However, the role of post-damage histone ubiquitylation in maintaining genomic integrity remains unclear.

### Sumoylation

In addition to ubiquitin, there are several ubiquitin-like proteins (UbLs). One of them is a small ubiquitin-related modifier (SUMO) polypeptide of <100 amino acids (11 kDa). SUMO is conjugated to a large number of cellular proteins, altering their interaction with other proteins, and it regulates intrinsic function or localization. More than 1,000 nucleoproteins undergo sumoylation (Hochstrasser 2009), and this pathway has been implicated in controlling many important processes, including regulation of the cell cycle, transcription, nucleocytoplasmic transport, DNA replication and repair, chromosome dynamics, and apoptosis, as well as ribosome biogenesis (Wang and Dasso 2009). SUMO has been shown to covalently attach to substrate proteins to form an isopeptide bond between a glycine in the UbL and a lysine residue in the substrate. The enzymatic cascade (E1-E2-E3) is similar to that involved in ubiquitylation, but it is a separate pathway. In mammalian cells, specific proteases called SENP (sentrin-specific peptidases) remove C-terminal residues, then the E1-activating enzyme (a heterodimer of SAE1/SAE2) activates SUMO, which is subsequently passed to the active site of the E2-conjugating enzyme (UBC9). UBC9 catalyzes the conjugation of SUMO to substrates by the formation of an isopeptide bond between the C-terminus of SUMO and the amino group of the target lysine (Iñiguez-Lluhí 2006). This step is enhanced by E3 ligases that interact with both the E2 and the substrate, thereby increasing the efficiency of SUMO transfer. Sumoylation is a dynamic and reversible modification, and specific isopeptidases are able to release the SUMO moiety. Post-translational modification by SUMO, unlike ubiquitin, has not been associated with protein degradation. To date, three different SUMO proteins have been described in vertebrates: SUMO-1, SUMO-2, and SUMO-3 (Shiio and Eisenman 2003). SUMO-1 shares 18 % identity with ubiquitin and shows similarity in the three-dimensional structure (Melchior 2009). SUMO-2 and SUMO-3 cannot be distinguished due to their <95 % homology; thus, they are often referred to as SUMO2/3. Surprisingly, the fourth SUMO protein is encoded in the human genome. SUMO-4 seems to be uniquely expressed in the spleen, lymph nodes, and kidney (Guo et al. 2004; Galisson et al. 2011). Bohren et al. (2004) have discovered that the expression level of SUMO-4 is the highest in the kidney.

Histone sumoylation was first described by Shiibo and Eisenman in 2003. They found that histone H4 can be modified by SUMO family proteins both in vitro and in vivo (Shiio and Eisenman 2003). These authors suggested that histone sumoylation causes the repression of transcriptional activity through the recruitment of HDAC1 and HP1. Nathan et al. (2003) provided evidence that all four core histones are sumoylated in budding yeast. Interestingly, the histone variant H2A.Z associated with active transcription (it localizes in gene promoters and genes near silenced regions) displays lower levels of SUMO compared with canonical H2A (Nathan et al. 2003). The sumoylation sites of the lysine residues of H2A (K126), H2B (K6, K7, K16, and K17), and H4 (K5, K8, K12, K16, and K20) were revealed by mass spectroscopy analysis (Nathan et al. 2003).

Sumoylation can also compete with other lysine-targeted modifications, including acetylation or ubiquitylation (Johnson 2004; Iñiguez-Lluhí 2006), and thereby can switch transcription from the active to the repressed state. It was demonstrated that the reduction of histone sumoylation results in increased histone acetylation (Nathan et al. 2006). In contrast, another study has shown that H4 sumoylation increases in parallel with H4 acetylation (Shiio and Eisenman 2003).

It appears that histone sumoylation serves as a transcription repressor and helps to maintain low basal levels of gene expression. There are a number of possible mechanisms by which SUMO promotes transcriptional repression. One possibility is that histone sumoylation may recruit HDACs (specifically class II HDACs) to deacetylate nucleosomal histones. A second mechanism could rely on HDAC activation by SUMO because it was observed that lowered HDAC sumoylation may indirectly lead to higher histone acetylation (David et al. 2002; Cheng et al. 2004).

In *Saccharomyces cerevisiae,* a higher level of H2B-SUMO was observed at telomeres than at more internal chromosomal sites, which may suggest the participation of histone sumoylation in telomeric silencing (Nathan et al. 2006). It is unclear whether sumoylation directly alters nucleosomal structure or packing and whether it promotes or inhibits interactions with non-histone proteins. Genomic analysis of SUMO-dependent changes in chromatin structure is very complex because many of the enzymes that regulate histone modifications (e.g., HATs and HDACs) can be sumoylated. Additionally, sumoylation of proteins that belong to the complexes interacting with DNA modification machinery (e.g., IκBα and PNCA, proliferating cell nuclear antigen, that interacts with DNMT1) and chromatin-remodeling complexes (e.g., RSF1, remodeling and spacing factor 1) may influence the epigenetic background (Nathan et al. 2003; Galisson et al. 2011). In summary, histone sumoylation is an important, dynamic modification that seems to play an essential role in chromatin structure and function.

### Biotinylation

Biotin is a B vitamin that is also referred to as vitamin H or vitamin B7. Cellular uptake of free biotin is mediated by the sodium-dependent multivitamin transporter (SMVT) (Wang et al. 1999). Biotin is a cofactor for four carboxylases, which play essential roles in the metabolism of glucose, proteins, and fatty acids (Camporeale and Zempleni 2006). Additionally, biotin is involved in gene regulation and chromatin structure (Zempleni et al. 2008). Biotinylation of histones is a reversible process, and it relies on the covalent attachment of biotin to the ε-amino group of lysine residues in core histones (Kothapalli et al. 2005). Two biotinyl ligases are involved in this process: biotinidase (BTD) (belonging to the nitrilase superfamily) (Brenner 2002) and holocarboxylase synthetase (HCS) (called biotin-dependent carboxylase) (Narang et al. 2004). Biotinidase uses biocytin (biotinyl-*q*-lysine) as a substrate (Hymes et al. 1995), whereas HCS uses biotin and ATP for biotinylation of histones (Narang et al. 2004). Hymes et al. (1995), based on in vitro study, proposed that biotinidase mediates the enzymatic catalysis of histone biotinylation. In contrast, the immunofluorescence studies revealed that biotinidase is localized in the cytoplasmic organelles but not the nucleus of human fibroblasts and Hep G2 cells (Stanley et al. 2004); hence, its role in histone biotinylation may be controversial. However, different results were reported in several other studies, in which the nuclear localization of biotinidase was confirmed (Pispa 1965; Chew et al. 2006a, b). In addition, all the lysine residues in histone H2A are targets for biotinylation by biotinidase (Chew et al. 2006a, b). Recent studies demonstrated that HCS has histone biotinyl ligase activity (Bao et al. 2011), and biotinylation of histones is mediated preferentially by HCS (Camporeale et al. 2006). Although debiotinylation of histones occurs, the exact mechanisms are largely unknown. It has been suggested that biotinidase might catalyze the debiotinylation of histones (Ballard et al. 2002). Presumably, alternate splicing of biotinidase might determine whether biotinidase acts as biotinyl histone transferase or histone debiotinylase (Zempleni 2005).

To date, eleven distinct histone biotinylation sites have been identified: five in histone H2A (K9, K13, K125, K127, and K129) (Chew et al. 2006), four in histone H3 (K4, K9, K18, and perhaps K23) (Kobza et al. 2005, 2008), and two in histone H4 (K8 and K12) (Camporeale et al. 2004). Other preliminary evidence indicates new biotinylation sites in histone H4, including K5 and K16 (Camporeale et al. 2004; Chew et al. 2006).

A growing body of evidence suggests that histone biotinylation plays an important role in biological processes, including gene silencing, chromatin remodeling, cellular responses to DNA damage, genome stability, mitotic condensation of chromatin (Kothapalli and Zempleni 2005; Rodriguez-Melendez and Zempleni 2003), and cell proliferation (Filenko et al. 2011). Pestinger et al. (2011) reported that H3K9bio, H3K18bio, and H4K8bio are enriched in pericentromeric heterochromatin and long tandem repeat regions (LTRs), but depletion of these marks at the IL-2 promoter correlates with transcriptional activation. A great abundance of H4K12bio was noticed in alpha-satellite repeats in pericentromeric regions (Camporeale et al. 2007), telomeric repeats (Wijeratne et al. 2010), and LTRs (Chew et al. 2008). H4K12bio represses transcription of LTRs (Chew et al. 2008), interleukin-2 (*IL*-*2*) (Camporeale et al. 2007), and the *SMVT* gene (Gralla et al. 2008).

It is estimated that approximately 30 % of histone H4 molecules in telomeric repeats are biotinylated at position K12 (Hassan and Zempleni 2008). A recent study showed that K12 biotinylation in histone H4 alters the structure of the nucleosomes and leads to <15 % increase in the amount of DNA wrapped around nucleosomes (Filenko et al. 2011).

The enrichment of H4K12bio depends on the concentration of biotin in the cell culture medium (Zempleni et al. 2009). Likewise, biotin supplementation in healthy human adults increased the relative enrichment of H4K12bio in the LTRs in primary peripheral blood mononuclear cells (Chew et al. 2008). Interestingly, LTR transcripts were increased when the enrichment of H4K12bio decreased due to biotin-deficit or HCS knockdown (Chew et al. 2008). An HCS knockdown disturbs gene regulation and decreases stress resistance and lifespan in *D. melanogaster*; it may be mediated by changes in chromatin modification (Camporeale et al. 2006). Bao et al. (2011) have found a direct physical interaction between human HCS and histone H3 causing subsequent biotinylation of lysines (K9 and K18) in its N-terminal region. Furthermore, the latter study revealed that HCS also strongly interacts with histone H4 but much less with histone H2A and H2B (Bao et al. 2011). Because HCS does not contain a DNA-binding motif that could direct it to distinct regions in chromatin, DNA sequence, biotin, chromatin marks and proteins, or RNA may be involved in targeting (Bao et al. 2011).

Other histone marks, including acetylation, phosphorylation, and methylation, influence histone biotinylation. Various modifications of histones can influence each other in synergistic or antagonistic ways. For example, acetylation of lysine and phosphorylation of serine residues decrease biotinylation of adjacent lysines in histone tails (Camporeale et al. 2004). In contrast, di-methylation of arginine residues enhances biotinylation of adjacent lysine residues (Kothapalli et al. 2005). Moreover, mass spectrometry revealed co-occurrence of biotinylation, acetylation, and mono-methylation in the same histone tail. It was observed that H4K8bio may be mono-methylated at K5 and that H4K12bio may be acetylated at K5 and K8 and mono-methylated at K16 (Chew et al. 2006). H4K12bio is a characteristic mark for repeat regions and heterochromatin areas, and it co-localizes with the repression mark H3K9me2 (Camporeale et al. 2007). Interestingly, if H4K12bio is decreased in biotin-deficient or HCS knockdown cells, the enrichment of H3K9me2 at the LTRs decreases substantially (Camporeale et al. 2007; Chew et al. 2008; Gralla et al. 2008). Preliminary observations indicate that HCS physically interacts with a histone H3 K9-methyltransferase (Zempleni et al. 2008). Furthermore, it was shown that repression of LTRs and other gene loci depends on an interaction between H4K12bio and DNA methylation. The enrichment of H4K12bio in LTRs was reduced by ~50 % in cells treated with 5′-azacytidine (a cysteine methylation inhibitor) (Chew et al. 2008). According to Chew et al., it is possible that methylcytosine-binding proteins (e.g., MeCP2) direct HCS to methylated DNA leading to local biotinylation of histone H4.

It seems that nutrient-dependent repression marks (cytosine methylation, H4K12bio, H3K9me2) synergize in the repression of LTRs. Taken together, at least three epigenetic modifications of DNA and histones are directly dependent on the vitamins biotin, folate, and niacin (Kirkland et al. 2007). Thus, biotin is emerging as an important dietary micronutrient for transcription regulation and chromatin remodeling and function. Even small alterations in the biotinylation of histones might be physiologically meaningful.

### ADP-Ribosylation

ADP-ribosylation is a reversible covalent PTM in which the ADP-ribose moiety from the co-substrate nicotinamide adenine dinucleotide (NAD+) is transferred to a specific amino acid of an acceptor protein. The process is mediated by members of the ADP-ribosyltransferase (ART) family. This family of enzymes is divided into two subclasses: first, ARTCs, extracellular proteins with sequence homology to clostridial C2 and C3 toxin (formerly known as membrane-associated ecto-ARTs), and second, ARTDs, proteins with distant sequence homology to bacterial diphtheria toxin (previously known as PARPs) (Messner and Hottiger 2011). ADP-ribosylation exists in two distinct forms, mono- and poly-ADP-ribosylation. The transfer of a single ADP-ribose residue is called mono-ADP-ribosylation, and subsequent attachment of the additional moieties generates polymeric ADP-ribose (PAR) chain structures resulting in poly-ADP-ribosylation. The best known ADP-ribosylated residues in eukaryotic cells are lysine (K), arginine (R), glutamate (E), aspartic acid (D), cysteine (C), asparagine (N), and phosphoserine (Hassa et al. 2006). Two classes of enzymes are capable of performing de-ADP-ribosylation, three ADP-ribosyl hydrolases (ARHs) and one poly-(ADP-ribose) glycohydrolase (PARD) (Koch-Nolte et al. 2008).

Surprisingly, mono-ADP-ribosylation is mostly found outside the nucleus, but poly-ADP-ribosylation occurs almost exclusively on the nuclear proteins (Hilz 1981). ADP-ribosylation plays a critical role in physiological and pathological cellular processes. Mono-ADP-ribosylation participates in the regulation of cell–cell and cell–matrix interactions, as well as in immune function (Corda and Di Girolamo 2002; Hassa et al. 2006). Poly-ADP-ribosylation, in turn, is engaged in the control of many crucial features, including cell differentiation, transcription, chromatin modification, DNA damage detection and repair, apoptosis, and carcinogenesis (Masutani et al. 2005; Hassa et al. 2006). It has been observed that histone proteins can also be mono- and poly-ADP-ribosylated; however, it is difficult to identify specific amino acid residues in histones due to the small amount (less than 1 %) of ADP-ribosylated histones in the total fraction (Hottiger 2011; Messner and Hottiger 2011). The linker histone H1 and core histones might be ADP-ribosylated in the cytoplasm during their synthesis or transport into the nucleus or after their incorporation into chromatin (Messner and Hottiger 2011). Most research focuses on the modification of nuclear histones; however, one study has described poly-ADP-ribosylation of histone H3 and H4 in the cytoplasm (Alvarez et al. 2011). All five histone proteins can be modified in vitro by ARTDs (Burzio et al. 1979). In native chromatin, histone H1, followed by H2B, is the major PAR acceptor, whereas other histones are weakly modified (Huletsky et al. 1989). Considering the role of histone H1 in the compaction of chromatin into higher-order structure, ADP-ribosylation of H1 is emerging as a modification that may decrease chromatin condensation. An ADP-ribosyl group is larger than other modifications and could induce changes in chromatin structure.

Previous studies have revealed a few ADP-ribose acceptor sites in histones, i.e., glutamate (at position E2 in H2B and at positions E2, 14, and 116 in H1) and a lysine residue (at position K213 in H1) (Burzio et al. 1979; Ogata et al. 1980a, b). However, these findings have not been confirmed by mass spectrometry. More recently, specific lysine residues were identified in in vitro experiments as PAR acceptor sites in core histones, and they include H2A (K13), H2B (K30), H3 (K27 and K37), and H4 (K16) (Messner et al. 2010). The reactions were catalyzed by ARTD1. It is worth noticing that the same lysines in histone H3 and H4 or neighboring amino acids are also acetylated and/or methylated. Thus, ADP-ribosylation could compete and interact with these modifications. In fact, an in vitro study has shown an inhibition of ADP-ribosylation of histone H4 mediated by acetylation of lysine 16 in histone H4 (Messner et al. 2010). The order of events in histone modifications seems to be crucial, e.g., mono- or poly-ADP-ribosylation of histones reduces their phosphorylation but not vice versa (Messner and Hottiger 2011). Additionally, it was found that ARTD1 prevents demethylation of H3K4me3 through ADP-ribosylation and inhibition of the histone lysine demethylase 5B (KDM5B) (Krishnakumar and Kraus 2010). Recently, cross-talk between H1.4 methylation and ADP-ribosylation has been described (Kassner et al. 2013). ARTD1-dependent PARylation of histones inhibits their subsequent methylation by SET7/9.

One of the most thoroughly investigated enzymes of the ARTD family is ARTD1 (abundant chromatin-associated nuclear protein); its induction by high amounts of NAD+ leads to chromatin relaxation (Kim et al. 2004). It was reported that ARTD1 is involved in the induction of local chromatin decondensation through poly-ADP-ribosylation of histone H1 (Meyer-Ficc et al. 2011). During DNA damage, the level of poly-ADP-ribosylation increased due to ARTD1 activation. DNA strand breaks are recognized and bound by ARTD1 resulting in the activation of the catalytic domain at the C-terminus (Langelier et al. 2008). Interestingly, ARTD1 activated by oligonucleotides is able to modify all five individual histones in in vitro assays (Messner et al. 2010). The benefit to the cell resulting from poly-ADP-ribosylation relies on the introduction of high negative charges in the histone molecules, which diminishes intrinsic histone–DNA interactions. Another ARTD enzyme, ARTD3, is able to modify histone H1.2 in vitro (Ruten et al. Rulten et al. 2011). Mono-ADP-ribosylation of core histones and H1 was primarily characterized in non-dividing cells, but dividing cells contain both mono- and poly-ADP-ribosylated histones (Boulikas 1990). It seems that poly-ADP-ribosylation is essential for replication because inhibition of this process arrests the growth of cells (Kidwell and Burdette 1974). Poly-ADP-ribosylated histones in proliferating cells might be generated at the replication fork due to the activation of ARTDs by unligated Okazaki fragments (Boulikas 1990). ADP-ribosylation is associated with transcriptionally active regions, for example, differentiating rat astrocytes and neuronal cultures exhibit high PAR levels (Chabert et al. 1992). Further analyses have shown that ARTD1 is enriched at active promoters and most likely excludes histone H1 from a subset of these promoters, which would suggest interplay between ARTD1 and H1 (Kraus 2008; Krishnakumar et al. 2008). It is still unknown whether mono- and poly-ADP-ribosylation participate to the same extent in the histone code. A recent study has revealed that ARTD1 activation is needed for long-term neuronal plasticity in mice (Goldberg et al. 2009). Fontan-Lozano et al. (2010) have shown that ARTD1 activation promotes histone H1 poly(ADP)-ribosylation and its release from promoters of specific genes regulated by the cAMP response element-binding protein (CREB) and that nuclear factor-κB (NF-κB) is required for memory consolidation. In *Drosophila*, ARTD1 was identified as being necessary for the chromatin decondensation in the *Hsp70* gene and the rapid disruption of the nucleosome structure (e.g., eviction of H3 and H4) (Petesch and Lis 2008). Nucleosome displacement or even eviction could be facilitated by histone poly-ADP-ribosylation.

Our knowledge of ADP-ribosylation is limited, and there are still many unanswered questions. Among them, is ADP-ribosylation of histone lysines a long-term modification that may be inherited as a stable epigenetic mark? Nonetheless, histone ADP-ribosylation is an interesting modification because, together with acetylation, methylation, and phosphorylation, it may constitute an epigenetic code.

### Crotonylation

Recently, lysine crotonylation (Kcr), a novel post-translational modification of histones, has been discovered (Tan et al. 2011). The crotonyl group (C_4_H_5_O) is most likely transferred from crotonyl-CoA to the ε-amino group of a target lysine residue. Tan et al. have identified 28 Kcr sites in human cells in the N- and C-terminal domains as well as the globular domains of the linker histone and four core histones. Lysine crotonylation is an evolutionarily conserved histone modification present in eukaryotic cells from yeast to human. It was found that the crotonylation mark is associated with active chromatin and is enriched at the promoters and enhancers of active genes in human somatic cells. Tan et al. (2011) have also shown that Kcr marks testis-specific genes on the sex chromosomes during spermatogenesis in mice. The authors suggested that histone Kcr can affect chromatin structure and facilitate histone replacement thereby influencing gene expression. In addition, a gain in histone Kcr in postmitotic male germ cells allows them to escape sex chromosome inactivation.

It is still not known what enzymes are responsible for crotonylation. Other questions are: what effects does histone Kcr have on the chromatin structure and function, and what proteins recognize and bind to crotonyllysine? Further studies are required to answer these questions.

### Proline Isomerization

Isomerization is the process by which a compound is converted into isomeric forms, i.e., forms with the same molecular composition but with a different arrangement of atoms in space. Isomers have different structures or configurations and, hence, usually differ significantly in physical and chemical properties. Isomerization of proteins has been known since 1968 (Tanford 1968), but histone isomerization was only reported in 2006 (Nelson et al. 2006). Although isomerization occurs spontaneously, several enzymes accelerate the interconversion of proline isomers. Proline isomerases can be divided into three families: the parvulins (Pin1 family), cyclophilins, and FK506-binding proteins (FKBPs) (Gothel and Marahiel 1999).

Nelson et al. (2006) identified proline isomerization, a new reversible and noncovalent histone modification. It has been shown that proline in the N-terminal tail of histones can adopt two distinct conformations, *cis* or *trans*, which affect the secondary structure of the tail. In this study, the proline isomerase Fpr4 (a member of the FKBPs) in *S. cerevisiae* was identified. Fpr4 binds to the N-terminal tails of histones H3 and H4 in vitro and catalyzes the isomerization of proline residues at position P30 and P38 in histone H3. Proline 38 is localized in proximity to lysine residue K36, which is methylated by Set2; therefore, cross-talk between proline isomerization and lysine methylation is possible. The proline isomerase Fpr4 controls the *cis*↔*trans* equilibrium at P38 of histone H3. The *cis* conformation at this position brings the tail closer to DNA and increases the opportunity for their interaction and nucleosome stability. Fpr4 catalytic activity may be needed for the formation of a higher-order chromatin structure (Nelson et al. 2006). Interestingly, Fpr4 inhibits Set2 methylation of H3K36 in vitro, while Fpr4 removal results in an increase in the trimethylation of K36 in vivo. Another group has suggested that di-methylation of K36 in histone H3 is independent of H3P38 (Youdell et al. 2008). In turn, methylation of H3K36 prevented the isomerization of H3K36 in vitro, and trimethylated H3K36 inhibited Fpr4 and favored active chromatin. Proline in the *cis* conformation reduces methylation by Set2, whereas the *trans* isomer facilitates Set2 lysine methylation. It is assumed that the *cis* conformation changes the secondary structure of the H3 tail, so that H3K36 no longer fits the active site of Set2 and the methyl K36 is more accessible for the demethylase JMJD2. The *trans* conformation of H3P38 is recognized by Set2 and creates favorable conditions for efficient methylation of K36. These results reinforce the idea that the catalytic activity of Fpr4 controls the methylation of H3K36 via isomerization of H3P38.

The latter results were derived from experiments carried out on yeast; in mammalian cells, an interaction between the phosphorylation of H3S28, the methylation of H3K27, and the isomerization of H3P30 was also reported (Nelson et al. 2006). Histones H2A and H2B are also interesting because they have multiple proline residues in proximity to modifiable amino acids. Among the many proline isomerase enzymes, hFKBP25 is an interesting human ortholog of yeast Fpr4, previously described as a high-affinity receptor for rapamycin (Jin and Burakoff 1993). It has been demonstrated that hFKBP25 co-immunoprecipitates with HDAC1 and HDAC2 (Yang et al. 2001). How the isomerization of histone prolines contributes to transcriptional and epigenetic regulation in humans is still to be deciphered.

### Aspartic Acid Isomerization

Aspartic acid (Asp) isomerization is a spontaneous conversion that can occur under physiological pH and temperatures in the absence of any enzymes. However, protein-l-isoaspartate *O*-methyltransferase (PIMT, also known as l-isoaspartyl protein carboxyl methyltransferase) was identified, in both prokaryotes and eukaryotes, as a repair enzyme that initiates the conversion of isoaspartic acid (isoAsp) to aspartic acid (Asp) (Clarke 1985). This enzyme catalyzes the transfer of a methyl group from SAM to the free carboxyl groups of isoAsp. The enzymatic methyl esterification of abnormal residues leads to their conversion to the normal form. These atypical Asp residues may perturb protein activity and lead to disruption of cellular functions. The amount of isoAsp increases under cellular stress and/or apoptosis (Cimmino et al. 2008; Doyle et al. 2013).

Aspartic acid isomerization seems to be the candidate for a new post-translational modification found recently at position D25 in histone H2B (Doyle et al. 2013), but no isoAsp acid was observed in other core histones (Young et al. 2001). The high level of isoAsp was noticed when the PIMT repair system was blocked, for example, by a knockdown of the PIMT gene in mice (Young et al. 2001). In mammals, PIMT transcripts were expressed predominantly in brain and testis, and it appears that neurons need tighter control of isoAsp than other cells (Mizobuchi et al. 1994).

The selective accumulation of isoAsp in histone H2B makes the histone immunogenic. Significantly higher amounts of antibody against both Asp and isoAspH2B_21–35_ in patients with systemic lupus erythematosus than in healthy controls have been observed (Doyle et al. 2013). In this regard, H2BDiso is emerging as an important factor in the development of autoimmune disease.

### N-formylation

N^6^-formylation is a noncanonical, endogenous secondary modification that arises from products of DNA oxidation in cells (Jiang et al. 2007). The proximity of histone lysine to DNA can facilitate the reaction of deoxyribose oxidation products with histone proteins. Thus, the oxidative and nitrosative stress in cells may have an effect on epigenetic mechanisms governing chromatin states. *N* ^6^-formylation was detected on both histone and non-histone nuclear proteins at relatively high levels. The formyl moiety of 3′-formylphosphate residues acylate the *N* ^6^-amino groups of lysine (Jiang et al. 2007). The core and linker histones are formylated in the tails and globular domains. The N^ε^-formyllysine residue is chemically similar to *N* ^6^-acetyllysine and may mimic lysine acetylation and interfere with normal histone modifications. Wiśniewski et al. (2008) have identified 19 formylation sites in all four core histones; H3K64, H4K79, and H2BK34 are involved in DNA binding or nucleosomal organization. In addition, some of the modifications occur in both acetylated and methylated forms, and these modifications can compete with each other (Wiśniewski et al. 2008). The linker histone H1 is the most frequently formylated histone. For variant H1.4, eleven formyllysines were mapped and three of them (K64, K85, and K97) are engaged in DNA binding.

It is suggested that lysine formylation could accumulate with age due to the slow turnover rates of histones, and this could contribute to the deregulation of chromatin function. Moreover, lysine formylation is promoted by oxidative stress and so may be involved in the development of stress-related diseases, including cancer.

### Propionylation and Butyrylation

Two novel PTMs, lysine propionylation and butyrylation, were discovered in vivo on histone H4 (Chen et al. 2007). Chen et al. (2007) have identified two histone acetyltransferases, p300 and CBP (CREB-binding protein), that use propionyl-CoA or butyryl-CoA as substrates to catalyze propionylation or butyrylation of lysine residues in vitro. Short-chain CoAs, including propionyl-CoA and butyryl-CoA, are structurally similar to acetyl-CoA and are present at high concentrations in cells. Propionyl-CoA is derived from odd-chain fatty acid catabolism and branched-chain amino acid oxidation, whereas butyryl-CoA is a metabolic intermediate formed during the β-oxidation of fatty acids, and it is a substrate for fatty acid elongation (Chen et al. 2007). The concentration of short-chain CoAs depends on the diet and cellular physiological conditions (King and Reiss 1985). Propionylation and butyrylation may be associated with cellular metabolic status and could regulate genes involved in energy metabolism.

An in vivo study has revealed lysine propionylation at K5, K8, and K12, as well as lysine butyrylation at K5 and K12 of histone H4 (Chen et al. 2007). Three of these lysine residues of histone H4 are also acetylated, while one (K12) is also methylated. Nano-HPLC/mass spectrometric analysis was also used to map in vitro lysine residues modified by HATs. The results indicated that lysine residues, including K5, K8, K12, K16, K31, K44, K77, K79, and K91, were both propionylated and butyrylated (Chen et al. 2007). Interestingly, HATs do not differentiate between acetyl-, propionyl-, and butyryl-CoA, so that the abundance of the donor substrate may determine the type of modification (Chen et al. 2007; Vollmuth and Geyer 2010). These modifications seem to be reversible because it has been demonstrated that HDACs (i.e., Hst2, Sirt1, Sirt2, and Sirt3) can catalyze efficient depropionylation and debutyrylation in vitro and in vivo with varying catalytic efficiency(Smith and Denu 2007; Garrity et al. 2007).

Liu et al. (2009) provided the first evidence for the existence of propionylation at H3 lysine 23 in mammalian cells. These authors also demonstrated that histone acetyltransferase p300 can catalyze this modification, whereas the HDAC Sir2 catalyzes the removal of the propionyl group in vitro (Liu et al. 2009). Another research group reported the detection of propionylation and butyrylation in the yeast histones H2A, H3, and H4, including H3K23 (Zhang et al. 2009). The presence of these two marks in a wide range of organisms suggests that they are evolutionarily conserved among eukaryotes.

The biological functions of lysine propionylation and butyrylation in histones remain unknown; however, propionyllysine or butyryllysine may be involved in the control of chromatin structure. These modifications neutralize the positive charge of lysine residues and may attenuate the histone–DNA interaction by neutralizing the positive charge of the nucleosomes, thereby exposing regulatory elements to transcription factors. Furthermore, it has been shown that H3K23prop and H3K14buty are recognized by chromatin “readers,” including bromodomain-containing protein 4 (Brd4), which provides a docking site to recruit a chromatin-remodeling enzyme (Vollmuth and Geyer 2010). It is believed that the presence of these modifications can block or promote the occurrence of another PTM at neighboring sites (Liu et al. 2009). Vollmuth and Geyer (2010) assumed that short-chain lysine *N*-acyl modifications, including acetylation, propionylation, and butyrylation, may indeed be regarded as linear analogs to tetrahedral mono-, di-, and trimethylation (Vollmuth and Geyer 2010). Undoubtedly, the discovery of lysine propionylation and butyrylation raises many interesting hypotheses; therefore, further analyses for better understanding their role in epigenetic control are needed.

## Nucleosome Level

### Nucleosome Positioning

Nucleosomes form the fundamental repeating unit of eukaryotic chromatin and play an important role in epigenetic regulation. They can limit DNA accessibility to cellular machinery through specific positioning of nucleosome core particles, which can be remodeled in an ATP-dependent manner (Zhang et al. 2008). Chromatin remodeling is required for transcriptional activity of genes and results in the alteration of accessibility to gene promoters and regulatory regions. Nucleosome positioning is a dynamic process that can be influenced by DNA sequence, histone variants and modifications, as well as chromatin remodeler complexes. There are currently four different ATP-dependent chromatin-remodeling families in eukaryotes: SWI/SNF, ISW, CHD, and INO80 (Clapier and Cairns 2009). In addition, the different histone modifications can be recognized by chromatin-modifying enzymes, suggesting a relationship between PTMs and nucleosome positioning. For example, histone-acetylated lysines are bound by Swi2/Snf2 (Mujtaba et al. 2007), while H3K4me3 is bound by CHD1 (Sims et al. 2005).

DNA methylation decreases the flexibility of DNA, resulting in shortening of the linker region and facilitating internucleosomal interactions (Correll et al. 2012). The position of one nucleosome influences the positioning of neighboring nucleosomes, creating open or closed chromatin structure. Nucleosome positioning has effects on DNA methylation. Depletion of histone H1 induces DNA hypomethylation, and thus H1 participates in the maintenance and/or establishment of specific DNA methylation patterns (Jin et al. 2011).

The composition of the nucleosome core octamer influences nucleosome positioning. A growing body of evidence has revealed that replacement of the histone H2A with H2A.Z may cause nucleosome sliding to a new stable position even without chromatin-remodeling proteins (Guillemette et al. 2005; Tolstorukov et al. 2009). In summary, it appears that all determinants may influence each other resulting in determined nucleosome positioning patterns.

## Histone Tail Clipping

Activation of gene transcription requires changes in the histone modifications associated with promoters. It is plausible that the histone tail clipping is another way to remove histone modifications and may influence local nucleosome positions. More than 30 years ago, it was found that in *Tetrahymena* the first six amino acids from the N-tail of histone H3 are removed (Allis et al. 1980). Further studies revealed that this type of activity also exists in yeast and mammals, but in these organisms, the first 21 amino acids of histone H3 carrying repressive marks are removed (Santos-Rosa et al. 2009; Duncan et al. 2008). In vivo, the H3 tail is clipped following the induction of transcription and preceding the process of histone eviction (Santos-Rosa et al. 2009).

There are many reports depicting the existence of histone proteolysis, but the proteolytic enzymes are not well characterized. A recent review article has presented a classification of histone proteases depending on pH and specificity (Purohit et al. 2012). In 1976, the histone H2A-specific protease activity was reported in chromatin from calf thymus (Eickbush et al. 1976). The proteolytic clipping of histones was also observed during mouse ESC differentiation. Cathepsin L, originally described as a lysosomal protease, was responsible for cutting histone H3 after the 21st residue from the N-terminus and the progressive removal of several residues (up to the 27th residue) (Duncan et al. 2008). The truncated H3 tail loses both active and repressive post-translational modifications. Mandal et al. (2012) provided earlier evidence of tissue-specific proteolytic processing of histone H3 in the nuclei of chicken liver. Their most recent study has indicated that glutamate dehydrogenase (GDH) can act as a histone H3-specific protease in chicken liver tissue (Mandal et al. 2013).

It is now apparent that the N-terminal and C-terminal tails of histones are susceptible to proteolysis, whereas the globular domains are relatively resistant to cleavage (Topping and Gloss 2011). Biswas et al. (2011) have reported that both histone H3 and H2A tail truncation destabilizes nucleosome structure. Moreover, truncation of the H2A C-terminal tail affects the binding of ATP-dependent chromatin-remodeling factors (Vogler et al. 2010) and may play an important role in nucleosome mobility. Interestingly, an in vitro study has shown that H4 or H2B tail truncation does not result in structural alterations in the nucleosome core (Biswas et al. 2011). Another in vitro experiment has shown that mutation or deletion of tail domains can cause transient unwrapping of DNA, changes in nucleosome sliding, and variation in the rate of H2A–H2B dimer exchange (Ferreira et al. 2007).

In conclusion, clipping of histone tails can be considered a new mechanism for removing histone modifications. In addition, clipping may influence nucleosome mobility and chromatin dynamics that could promote or inhibit transcription activity.

## Cross-Talk of Chromatin Marks

**Fig. 1:**
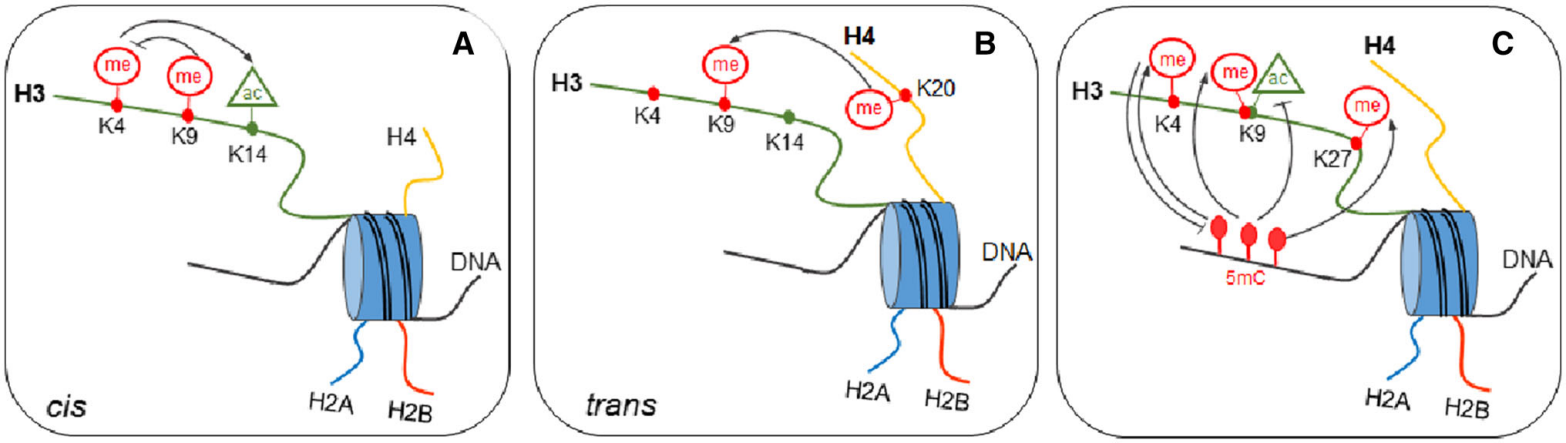
Cross-talk between chromatin marks. Intranucleosomal interaction: *cis* configuration—interaction between the modifications at the same histone tail (**a**) and *trans* configuration—interaction between the modification of the different histone tails (**b**). Intranucleosomal interaction between DNA methylation and histone modification (**c**)

Many studies have shown that the histone post-translational modifications can be influenced by neighboring PTMs and work in a coordinated manner. In 2000, Strahl and Allis proposed the histone code hypothesis, which states that “multiple histone modifications, acting in a combinatorial or sequential fashion on one or multiple histone tails, specify unique downstream function” (Strahl and Allis 2000). However, the relationships between chromatin marks, including DNA methylation and histone modifications, seem to be much more complex because these processes could mutually influence each other (Zhang and Reinberg 2001; Fischle et al. 2003). It has been proposed to define them as an”epigenetic code” that shapes the structure of chromatin and thus affects transcriptional activity. The cross-talk between different modifications can occur via diverse mechanisms. First, communication at the level of a single histone tail (the *cis* effect), for example, methylation on H3K9, can inhibit acetylation of the H3 tail and methylation of H3K4 (Fig. 1a) (Wang et al. 2001; Fischle et al. 2003). In addition, the identification of specific lysine residues as acceptor sites for several different modifications (e.g., acetylation, methylation, ADP-ribosylation, propionylation, and butyrylation) indicates direct competition for the same amino acid residues. Second, interactions at the level of nucleosomes mean that the modifications on different histones can affect each other (the *trans* effect). For example, trimethylation on H3K9 is required for the induction of H4K20 trimethylation (Fig. 1b) (An 2007). Third, DNA methylation and histone modification pathways can influence each other and establish the epigenetic landscape important for development, somatic cell reprogramming, and tumorigenesis. Relationships between DNA methylation and histone H3 methylation, particularly H3K4, H3K9, and H3K27, have been observed (Fig. 1c). There is also a strong anti-correlation between different DNA methylations; for example, the presence of the H3K4me mark prevents de novo methylation of CpG islands in the embryo (Cedar and Bergman 2009). The cross-talk between DNA methylation and histone modification most likely is mediated by SET domain HMTs and DNMTs (Cedar and Bergman 2009).

Previously, it was thought that the epigenetic code, especially DNA methylation and H3K9me, was stable, hereditary marks (Margueron and Reinberg 2010; Blancafort et al. 2013). Currently, we know that there are many enzymes that can “write,” “read,” and “erase” chromatin marks (Tables 3 and 4). It is doubtful that the epigenetic code is indeed irreversible. However, some of the epigenetic modifications pass down through generations and are responsible for the maintenance of cellular phenotypes, including reprogramming, imprinting, X chromosome inactivation in females, heterochromatin formation, and tissue-specific gene silencing. Chromatin marks also have a major role in various cellular processes, including replication, DNA repair, alternative splicing, and chromosome condensation. The epigenetic information stably transmitted through mitotic and meiotic cell divisions is crucial for the establishment of the genomic chromatin environment and is called the epigenetic memory.

**Table 3:** Writers and erasers in mammals

Modification	Writer	Eraser
DNA methylation	DNA methyltransferases: DNMT1, DNMT3A/3B	Enzymes of demethylation pathway: AID/APOBEC; DNA glycosylases: TDG, SMUF1, MBD4; TET family
Histone methylation	HMTs, lysine and arginine-specific	HDMs
Histone acetylation	HATs	HDACs
Histone phosphorylation	Kinases	Dephosphatases
Histone ubiquitination	Ubiquitin ligases	Isopeptidases
Histone sumoylation	Serin-specific peptidases (SENP)—remove C-terminal residue, ligases—catalyzed conjunction SUMO to lysine	Isopeptidases
Histone biotinylation	Biotinyl ligases: BTD, HCS	Debiotinidase: BTD?
Histone citrullination	Peptidylarginine deaminases: PADI2, PADI4	
Histone ADP-ribosylation	ADP-ribosyltransferases (ARTs)	ADP-ribosyl hydrolases (ARHs) poly-(ADP rybosylase) glycohydrolase (PARD)
Histone *β*-N-glycosylation	Transferase: *O*-linked *β*-*N*-acetylglucosamine transferase (OGT)	Hydrolase: *O*-linked *β*-*N*-acetylglucosaminidase (OGA)
Histone prolinę isomerization	Proline isomerase: FK506 binding proteins (FKBPs)	
Histone propionylation	Histone acetyltransferases: p300, CBP	HDACs
Histone butyrylation	Histone acetyltransferases: p300, CBP	HDACs

**Table 4:** Readers of epigenetic modifications

Recognition site	Reader domain	Protein	Modification
Methylcytosine	MBD	MBD1, MBD2, MBD4, MeCP2	5-methylcytosine (5mC)
	SRS	UHRF1, UHRT2	
	BTP/POZ-Zn-finger	Kasio, ZBTB4, ZBTB38	
	IPT/TIG	RBP-J	
Methyllysine	Chromobarrel (*R*)	MOF, Eaf3, MRG15,	H3K36me2/me3, H3K4me1, H4K20me1
	CG (*R*)	HP1, CDH1, PC, MPP8, CDY, CDYL, CDYL2, CBX7, MCL3,	H3K9me2/me3, H3K27me2/me3
	Tudor (R)	PHF1, PHF19, PHF20, TDRD7,	H3K36me3
	TTD (R)	53BP1, KDM4A, KDM4B, KDM4C, Sgf29, UHRF1	H3K4me3, H3K9me3, H4K20me2
	MBT (*R*)	CGI-73, L(3)HBTL, SFMBT, PHF20L1	H3K4me1, H3K9me1/me2, H3K20me1, H4K4me1
	PWWP (*R*)	DNMT3A, BRPF1, NSD1-3, MSH-6, N-PAC, Pdp1	H3K36me3, H4K20me1/me3, H3K79me3
	ADD (*Pt*)	DNMT3L	H3K9me3
	Ankyrin repeats	G9a/GLP	H3K9 me1/me2
	BAH	ORC1	H4K20me2
	DCD (*R*)	CHD1	H3K4me1/me2/me3
	PHD (*Pt*)	BHC80, BPTF, AIRE, RAG2, ING1-5, BPTF, TAF3, PHF2, PNF8,PHF13, PHF13, Pygo, YNG1, SMCX,	H3K4me3, H3K4me2, H3K9me3
	WD40	WDR5/WDR9, EED, LRWD1,	H3K27me3, H3K9me3
	zf-CW (*Pt*)	ZCWPW1	H3K4me3
Methylarginine	ADD (*Pt*)	DNMT3L	H4R3me2 s
	Tudor (*R*)	AKAPI1, TDRD2-3, TDRD5,TDRD8-10, SMN1, SPF30,	H3Rme2, H4Rme2
	WD40	WDR5	H3R2me2
Acetyllysine	BD	GCN5, PRBM1,	H3Kac, H4Kac, H2AKac, H2BKac
	DBD (*R*)	Rsc4, TAF1, Brdt,	H3KacKac, H4KacKac
	DFP (*Pt*)	DPF3b	H3K14ac
	PH	histone chaperone Rtt106	H3K56ac
Phosphoserine	14-3-3	14-3-3ξ, 14-3-3β, 14-3-3γ, 14-3-3η, 14-3-3ε, 14-3-3μ, 14-3-3θ	H3S10ph, H3S28ph
	tandem BRCT		H2A.XS139 (γH2AX)
Phosphothreonine	BIR		H3T3ph
Propionyllysine	BD	Brd4	H3K23
Butyryllysine	BD	Brd4	K3K14
Unmodified H3	ADD (*Pt*)	DNMT3L	unmodified histone H3
	PHD (*Pt*)	UHRF1	unmodified histone H3
	WD40	Nurf55	unmodified histone H3

*ADD* ATRX-DNMT3-DNMT3L, *BAH* bromo-adjacent homology, *BD* bromodomain, *CD* chromodomain, *DCD* double chromodomain, *DBD* double bromodomain, *DFP* double PHD finger, *MBD* methyl-CpG-binding domain, *MBT* malignant brain tumor, *PH* double pleckstrin homology, *PHD* plant homeodomain, *PWWP* Pro-Trp-Trp-Pro; *SRA* SET- and Ring finger-associated domain, *TTD* tandem Tudor domain, *zf-CW* zinc finger CW, *(Pt)* PHD-type, *(R)* Royal superfamily

**Fig. 2:**
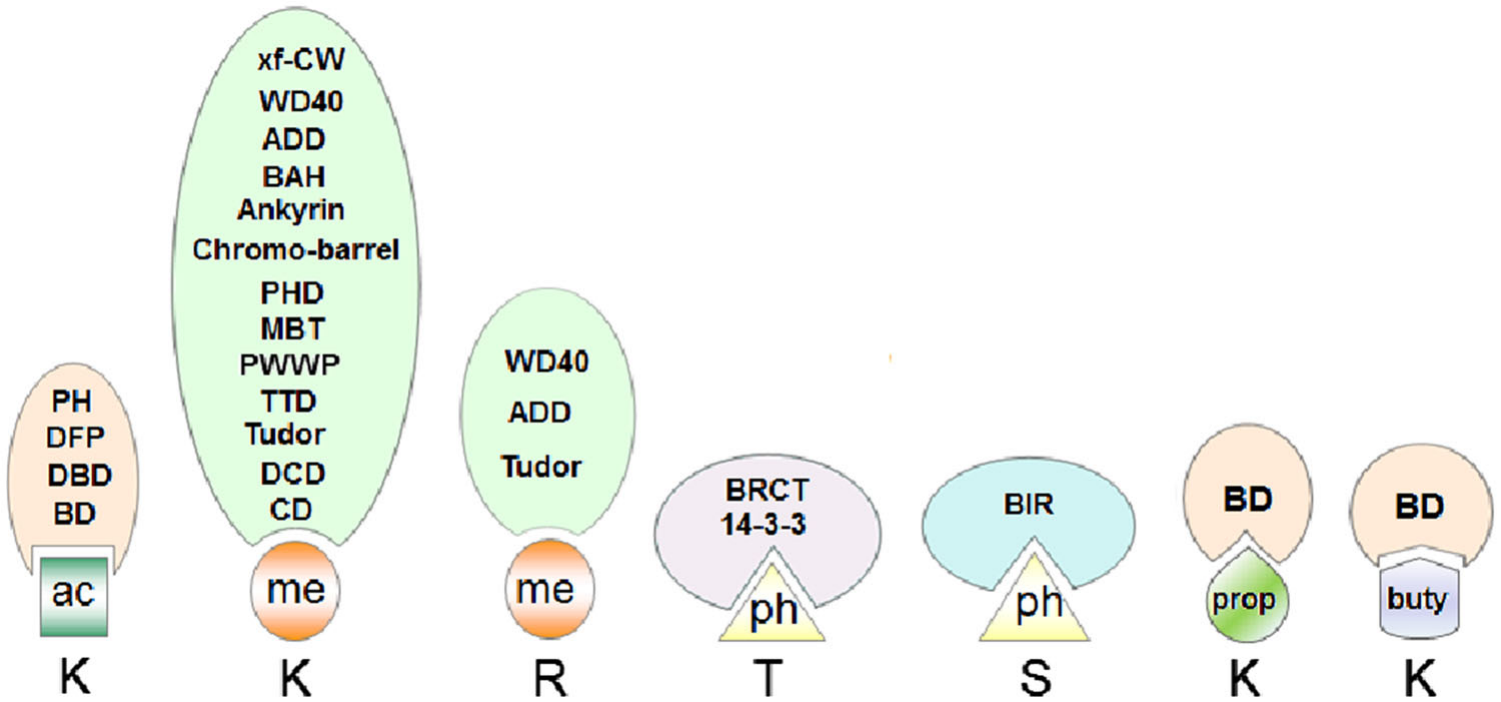
Protein domains capable of recognizing specific histone modifications. *Kac* acetylated lysine, *Kme* methylated lysine, *Tph* phospotylated threonine, *Sph* phosphorylated serine, *Kprop* propionylated lysine, *Kbuty* butyrylated lysine. For more abbreviations see Table 4

The list of newly identified histone readers has grown rapidly, given the extensive and complex nature of the chromatin landscape (Table 4). The direct or indirect interactions between “readers” or “writers” are essential for the cross-talk of various chromatin constituents. Different types of protein domains that recognize histone modifications have been identified (Fig. 2).

## Non-coding RNA

Genome-wide surveys have revealed that a large portion of the eukaryotic genomes is transcribed into non-coding RNA (ncRNA). Based on functional relevance, ncRNAs can be divided into two classes, structural and regulatory ncRNAs. Structural ncRNAs (or housekeeping ncRNAs) are generally constitutively expressed and are required for the normal function and viability of the cell. This group includes transfer RNAs (tRNAs), ribosomal RNAs (rRNAs), small nuclear (snRNAs), small nucleolar RNAs (snoRNAs), RNase P RNAs, and telomerase RNA (Prasanth and Spector 2007). In contrast, regulatory ncRNAs are expressed at certain stages of development, during cell differentiation, or as a response to environmental stimuli. Based on ncRNA length, regulatory ncRNA can be further divided into at least three groups: (1) short ncRNA including microRNA (miRNA) (22–23 nt) and piwi-interacting RNA (piRNA) (26–31 nt); (2) medium ncRNA (50–200 nt); and (3) long ncRNA (>200 nt) (Nie et al. 2012).

**Fig. 3:**
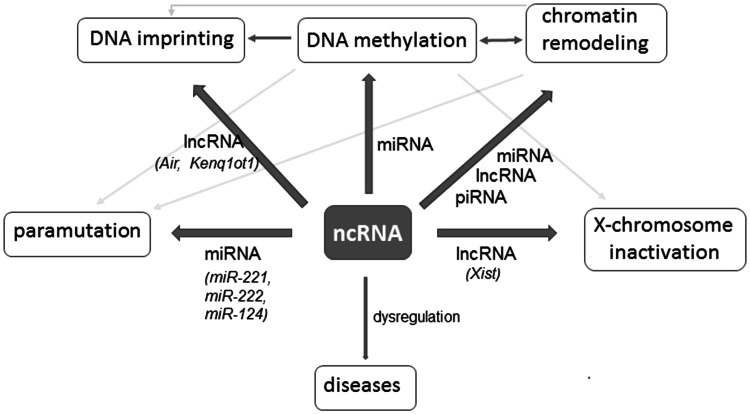
Schematic ncRNAs and chromatin regulatory network. ncRNAs influence different epigenetic events. Regulation involving miRNAs is the best known, particularly interesting is their participation in epigenetic heredity. miRNA-mediated inheritance is provided by the paramutation. Paramutation is an allelic interaction, one allele (called paramutagenic) causes heritable epigenetic changes in the second allele (called paramutable) of the same gene mediated by miRNA or siRNA. lncRNAs are also involved in epigenetic network, one of the first identified was *Xist*, the master regulator of X chromosome inactivation. *Air*, *Kenq1ot1*, *Xist*—the name of RNA genes

An increasing body of evidence suggests that ncRNAs can affect the expression of other genes at the level of transcription or translation and play a role in chromatin regulation via interaction with chromatin-modifying enzymes and transcription factors. Many studies have reported that miRNA, small interfering RNA (siRNA), piRNA lncRNAs, promoter-associated RNAs (paRNAs), centromere repeat-associated small interacting RNAs (crasiRNAs), and telomere-specific small RNAs (tel-sRNAs) are engaged in epigenetic regulation (for a review, see Kaikkonen et al. 2011; van Wolfswinkel and Ketting 2010). Schematic interaction between chromatin and some ncRNAs is depicted in Fig. 3.

### miRNAs

**Fig. 4:**
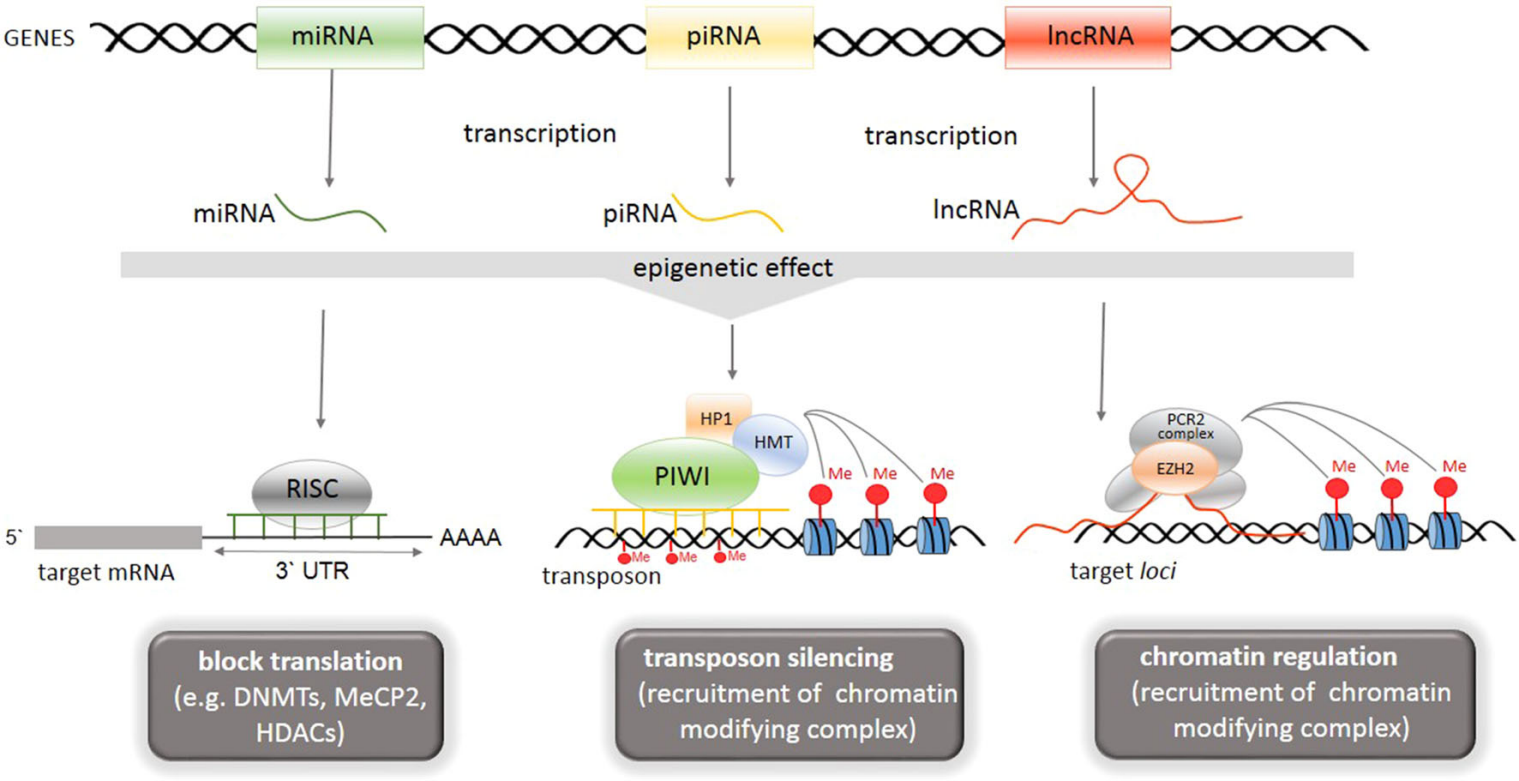
Effects exerted by ncRNA on the epigenetic regulations. Mature miRNAs after the incorporation into RISC complex bind to the complementary sequence in the 3′-UTR region of target transcript. miRNAs negatively regulate their targets by one of the four ways: (1) mRNA cleavage, (2) translation repression, (3) mRNA deadenylation, and (4) mRNA P-body localization. piRNA associated with PIWI proteins mediated in histone modifications and de novo DNA methylation. lncRNAs guide chromatin-remodeling complexes to specific site and also serve as scaffolds for modifying complexes

The miRNAs are the best known class of short ncRNAs, 19–29 nucleotides in length, that regulate gene expression at the post-transcriptional level. The miRNA molecules either cleave or repress translation of target mRNA resulting in decreasing levels of gene expression (Fig. 4). The group of miRNAs involved in epigenetic regulation is called “epi-miRNA” (Iorio et al. 2010). The miRNAs can influence epigenetic phenomena either by directly inhibiting enzymes involved in DNMTs, histone modifications, and chromatin remodeling (Table 5), or by altering the availability of substrates necessary for these enzymatic reactions. New evidence has indicated that small RNAs can play a key role in the paramutation mechanism and thus act as transgenerational signaling molecules.

**Table 5:** miRNAs regulating epigenetic pathway-related genes

MIRNA	Target	Role of target gene	Reference
DNA-modifying enzymes			
*miR*-*148*	DNMT1	DNA methylation	Duursma et al. (2008)
*miR*-*152*	DNMT1	DNA methylation	Denis et al. (2011)
*miR*-*301*	DNMT1	DNA methylation	Iorio et al. (2010)
*miR*-*126*	DNMT1	DNA methylation	Denis et al. (2011)
*miR148*	DNMT3B	DNA methylation	Denis et al. (2011)
*mi*-*29 family*	DNMT3A/3B	DNA methylation	Fabbri et al. (2007)
*miR*-*132*	MeCP2	Protect MeCp2 binding to DNA	Sato et al. (2011)
Transcription factors			
*miR*-*29b*	Sp1	Regulate DNMT1 transcription	Garzon et al. (2009)
*miR*-*290* cluster	Rbl2	Repressor of DNMTs transcription	Benetti et al. (2008), Sinkkonen et al. (2008)
*miR*-*K12*-*4*-*5p* virial	Rbl2	Repressor of DNMTs transcription	Iorio et al. (2010), Lu et al. (2010)
Chromatin remodelers			
*miR*-*29b/c*	YY1	Recruits PCR2 and HDAC to specific genome locus	Sato et al. (2011)
*miR*-*26a, miR*-*101, miR*-*205, miR*-*214,*	EZH2 (belongs to PRC1 complex)	PCR1 catalyzes ubiquitination of histone H2A, cooperate with PRC2	Sato et al. (2011)
*miR*-*128, miR*-*203*	Bim1 (belongs to PRC2 complex)	PCR2 facilitates histone methylation	Sato et al. (2011)
Histone-modifying enzymes			
*miR*-*449a*	HDAC1	Histone deacetylation	Liep et al. (2012)
*miR*-*1, miR*-*140*	HDAC4	Histone deacetylation	Liep et al. (2012)

Interestingly, inhibition of Dicer or Drosha (key enzymes in miRNA biogenesis) disrupts miRNA biogenesis and indirectly affects methylation patterns (Iorio et al. 2010; Liep et al. 2012). Loss of the *miR*-*290* cluster in Dicer-deficient mouse ESCs results in DNMT1, DNMT3A, and 3B downregulation corresponding to decreases in DNA methylation (Benetti et al. 2008; Sinkkonen et al. 2008). This miRNA family targets retinoblastoma-like 2 protein (*Rbl2*), which represses transcription of DNMTs (Benetti et al. 2008; Sinkkonen et al. 2008).

miRNAs can regulate the expression of genes that directly or indirectly regulate epigenetic status, so that when the miRNA-epigenetic regulatory circuitry is disrupted, normal chromatin function may be impaired leading to various diseases.

### Small Interfering RNAs (siRNAs)

Several independent laboratories have reported that siRNA-mediated suppression of transcription is associated with histone and DNA methylation of mammalian cells, which target the promoter region (Morris et al. 2004; Castanotto et al. 2005; Suzuki et al. 2005). However, Li et al. (2006) have shown that siRNA targeted to promoters of specific genes resulted in their re-expression The authors have postulated that the siRNA-mediated process did not change the state of DNA methylation, but it was associated with histone demethylation (Li et al. 2006). Therefore, siRNAs activate then repress transcription, and this phenomenon was confirmed by Chen et al. (2008) 2 years later.

### Antisense RNAs (asRNAs)

Antisense RNAs (asRNAs) are single-stranded RNAs complementary to mRNA that are involved in the mechanism of DNA methylation. It has been discovered that overexpression of Khps1, an endogenous antisense transcript, reduced demethylation of CG sites in the T-DMR (tissue-dependent differentially methylated region) (Mattick and Makunin 2005; Zhou et al. 2010).

### PIWI-Interfering RNAs (piRNAs)

PIWI-interfering RNAs (piRNAs) are the largest class of small ncRNAs in vertebrates, with a typical length of 25–33 nt. The piRNAs guide DNA methylation, and they maintain retransposon silencing during spermatogenesis in mouse germ cells (Lin 2007). PIWI–piRNA complexes play essential roles in the de novo DNA methylation of transposable elements in fetal, male germ cells (Zhou et al. 2010). Moreover, PIWI–piRNA complexes bind to numerous piRNA-complementary sequences in the *Drosophila* genome (Yin and Lin 2007). Huang et al. (2013b) have demonstrated that inserting piRNA-complementary sequences into an ectopic site leads to Piwi, HP1a, and Su(var)3-9 recruitment to this site, as well as H3K9me2/3 enrichment. These results indicate that piRNA is both necessary and sufficient to recruit PIWI and epigenetic factors to specific genomic sites (Fig. 4).

### Long Non-coding RNAs (lncRNAs)

LncRNA transcription and processing are complicated processes in which the majority of lncRNAs are spliced, polyadenylated, and 5′-capped (as in protein-coding RNA). In particular, a large group of lncRNAs is antisense to known protein-coding transcripts, so they are also referred to as natural antisense transcripts (NATs) (Nie et al. 2012). In recent years, the functions of only a few lncRNAs have been characterized. Unlike other ncRNAs, most lncRNAs are localized in the nucleus, which would suggest that they are involved in the regulation of chromatin. They most likely guide chromatin-modifying complexes to specific genomic loci (Fig. 4). Indeed, it has been demonstrated that lncRNAs recruit chromatin-remodeling complexes to specific chromatin loci in *cis* or *trans*. For example, lncRNAs, including *Air*, *Kcnq1ot1*, and *Evf*-*2,* target chromatin-modifying complexes to their target genes in *cis,* but *HOTAIR* directs the chromatin-modifying complexes PRC2 and LSD1 to gene loci *in trans* (Moran et al. 2012). Nuclear lncRNA molecules selectively interact directly or indirectly with the components of chromatin-remodeling complexes, including EZH2, SUZ12, CBX7, CoREST, and JARID1C/SMCX (Nie et al. 2012). Apart from chromatin remodeling, lncRNAs may be involved in epigenetic gene silencing, i.e., genomic imprinting and X chromosome inactivation (Ponting et al. 2009).

### Promoter-Associated RNAs (paRNAs)

A new class of ncRNAs derived from eukaryotic promoters defined as promoter-associated RNAs (paRNAs) were discovered. The length of paRNAs ranges from 22 to 200 nt, so they include short, medium, and long molecules. This class encompasses promoter-associated small RNAs (PASRs), terminal-associated short RNAs (TASRs), transcription start site-associated RNAs (TSSa-RNAs), transcription initiation RNAs (tiRNAs), and promoter-upstream transcripts (PROMTs) (Kaikkonen et al. 2011). Kapranov et al. (2007) identified short paRNAs (PASRs and TASRs) which are located near the promoter or transcription start side (TSS). TSSa-RNAs are situated within -250 to +50 of TSSs and flank active promoters in both sense and antisense directions. Similarly, PROMTs are located upstream of genes also in both directions (Preker et al. 2008), whereas tiRNAs are present in a greater density downstream of TSSs of highly expressed genes (Taft et al. 2009).

It is suggested that paRNAs contribute to transcriptional regulation and chromatin organization. For example, the repressive Polycomb group (PcG) protein complex binds to stem-loop structures of short RNAs and mediates transcriptional gene silencing (Kanhere et al. 2010). Furthermore, it was shown that the presence of a promoter-associated RNA at the promoter of human ubiquitin C gene led to long-term silencing which resulted from the increase in histone and DNA methylation (Hawkins et al. 2009). Furthermore, it was highlighted that PASRs, tiRNAs, and long paRNAs play a role in the maintenance of chromatin structure and activation of chromatin marks (for a review, see Sana et al. 2012).

### Centromer Repeat-Associated Small Interacting RNAs (crasiRNAs)

The crasiRNAs, 34–42 nt in length, are derived largely from repeated elements and are very important for centromere establishment as well as chromosome segregation. The crasiRNAs have been found in centromere protein A (CENP-A)-rich regions of the centromere and may comprise an integral component of epigenetic machinery necessary for heterochromatin formation (Carone et al. 2009). Although the mechanism by which these RNA molecules influence centromere function remains unknown, it is proposed that crasiRNAs facilitate the recruitment of chromodomain-like adaptor proteins to the centromere-specific DNA. This event triggers H3K9 methylation, HP1 interaction, and ultimately DNA methylation (Lindsay et al. 2012). Therefore, crasiRNAs could be considered as one of the regulatory elements in epigenetic phenomena.

### Telomere-Specific Small RNAs (tel-sRNAs)

The tel-siRNAs, approximately 24 nt long, are detected in telomere and subtelomere regions in mammalian cells. It was observed that the level of tel-sRNAs is down-regulated in cells that carry null mutation *Suv39h1/h2* ^−*/*−^, and thus it suggests that tel-siRNAs are a subject to epigeneitic regulation (Cao et al. 2009). On the other hand, tel-sRNAs contain UUAGGG repeats that inhibit telomerase activity in vivo (Schoeftner and Blasco 2008). Thus, tel-siRNAs could potentially act as a sensor of chromatin status and mediator in the telomeric length control and telomeric heterochromatin formation (Cao et al. 2009).

## Conclusions

Despite the fact that the mechanisms of epigenetic regulation have been studied for many years, there are still open questions. New interesting discoveries in the field of epigenetics appear each year. Currently, advanced high-throughput technologies allow for the exploration of multifaceted contacts between chromatin components, regulatory proteins, and the transcription machinery. Moreover, much progress has been made in the characterization of ncRNAs as an additional component of epigenetic machinery. It is known that the dysregulation of epigenetic control leads to many diseases affecting the brain, immune and cardiovascular systems, and diabetes, as well as cancers. Interestingly, certain epigenetic changes induced by environmental factors are responsible for some of these diseases. Some of them seem to be reversible, and they may be promising new targets for treatment. Unfortunately, epigenetic drugs currently used against cancer are not specific, but we firmly believe that an efficient, specific epigenetic therapy could be possible in the near future. A better understanding of the epigenetic network, particularly chromatin regulatory proteins, will help us develop innovative treatment strategies.
